# Simultaneous Quantification of Serum Lipids and Their Association with Type 2 Diabetes Mellitus-Positive Hepatocellular Cancer

**DOI:** 10.3390/metabo13010090

**Published:** 2023-01-06

**Authors:** Zhihong Yue, Lin Pei, Guangyan Meng, Aimin Zhang, Meng Li, Mei Jia, Hui Wang, Linlin Cao

**Affiliations:** 1 Department of Clinical Laboratory, Peking University People’s Hospital, Beijing 100044, China; 2 Department of Endocrinology and Metabolism, Peking University People’s Hospital, Peking University Diabetes Center, Beijing 100044, China

**Keywords:** hepatocellular cancer, serum lipids, type 2 diabetes mellitus, hexadecanedioic acid, 15-keto-13,14-dihydroprostaglandin A2, biomarker signature

## Abstract

Type 2 diabetes mellitus (T2DM) has been recognized as one of the most important and independent risk factors for hepatocellular cancer (HCC). However, there is still a lack of ideal tumor markers for HCC detection in the T2DM population. Serum lipids have been revealed as potential tumor markers for HCC. In this study, our objective was to develop a novel liquid chromatography-tandem mass spectrometry (LC-MS/MS) method to detect several lipids including 8,15-dihydroxy-5,9,11,13-eicosatetraenoic acid (8,15-DiHETE), hexadecanedioic acid (HDA), 15-keto-13,14-dihydroprostaglandin A2 (DHK-PGA2), ricinoleic acid (RCL), octadecanedioic acid (OA) and 16-hydroxy hexadecanoic acid (16OHHA) in serum and explore their diagnostic potential for T2DM-positive [T2DM(+)] HCC. A robust LC-MS/MS method was established for the measurement of 8,15-DiHETE, HDA, DHK-PGA2, RCL, OA, and 16OHHA. The methodology validation was conducted, and the results suggested the reliability of this LC-MS/MS method for targeted lipids. Several serum lipids, including 8,15-DiHETE, HDA, DHK-PGA2, and OA were increased in T2DM(+) HCC patients. A biomarker signature that incorporated HDA, DHK-PGA2, and AFP was established and showed good diagnostic potential for T2DM(+) HCC, and the area under the ROC curve (AUC) was 0.87 for diagnosing T2DM(+) HCC from T2DM individuals. Additionally, the biomarker signature diagnosed small-size (AUC = 0.88) and early-stage (AUC = 0.79) tumors with high efficacy. Moreover, the biomarker signature could differentiate T2DM(+) HCC from other T2DM(+) tumors, including pancreatic, gastric and colorectal cancer (AUC = 0.88) as well. In **conclusion,** our study develops a novel tool for early diagnosis of T2DM(+) HCC in T2DM patients.

## 1. Introduction

Hepatocellular cancer (HCC) is the most prevalent primary liver cancer, and it is the second most prevalent cause of cancer mortality all over the world [1,2]. During the past several decades, rapid improvement has been made in the knowledge of the epidemiology and etiology of HCC, and new methods for the prevention, monitoring, and early detection of HCC have been used in clinics as well [3]. However, the vast majority of newly diagnosed HCCs have already been at advanced stages. The potentially curative surgery is not appropriate for the advanced-stage HCC, whose prognosis is poorer than that of early-stage HCC [4,5]. Therefore, it is essential to monitor high-risk individuals for HCC appropriately and detect HCC tumors as early as possible.

One of the most important risk factors for HCC is type 2 diabetes mellitus (T2DM) [6,7]. T2DM is a chronic disease with progressive hyperglycemia, accompanied by dysregulated insulin secretion or insulin resistance, and accounts for over 90% of all diabetic cases [8]. The morbidity of diabetes in China has grown from 0.67% to over 10% during the past four decades, posing a serious threat to the health of the Chinese population [9]. It has been reported that the incidence and mortality of HCC are more than two-fold upregulated in T2DM patients [10,11]. Due to the huge number of T2DM patients, it is necessary to carry out suitable monitoring of the T2DM population to diagnose HCC as early as possible and improve the prognosis of HCC. However, there is still a lack of effective indicators to monitor the T2DM population for early detection of HCC. Serum biomarkers that are widely used, including serum alpha-fetoprotein (AFP) and des-gamma-carboxy prothrombin (DCP), are mainly related to advanced-stage HCC than early-stage disease, and would not be suitable for the diagnosis of early tumors [4,12]. Therefore, it is important to discover new indicators for HCC monitoring in T2DM individuals.

The liver is the most important center of metabolic processes, including lipid synthesis, storage, and oxidation, and HCC development damages the original biochemical function of the liver, which will inevitably lead to abnormal lipid metabolism [13,14]. Dysregulation of lipid metabolic pathways, such as fatty acid metabolism and biosynthesis of unsaturated fatty acids pathway, has been confirmed at the metabolite level in HCC, and serum lipid metabolites, such as arachidonic acid and 12-hydroxyeicosatetraenoic acid, are often altered in HCC patients [15,16,17,18]. However, most of these studies were concerned with HCC together with viral infections, including hepatitis B and C viruses, while there were no studies focusing on the dysregulated lipid metabolism of T2DM-positive [T2DM(+)] HCC. In our previous study, metabolomic analyses of the serum from T2DM and T2DM(+) HCC individuals were performed, and several serum lipids and lipid-like molecules were found to be altered in HCC [19]. However, the precise levels of these lipids in serum and their role in the surveillance and diagnosis of T2DM(+) HCC are largely unknown.

In the present study, a robust liquid chromatography-tandem mass spectrometry (LC-MS/MS) method for detecting several lipids simultaneously, including 8,15-dihydroxy-5,9,11,13-eicosatetraenoic acid (8,15-DiHETE), hexadecanedioic acid (HDA), 15-keto-13,14-dihydroprostaglandin A2 (DHK-PGA2), ricinoleic acid (RCL), octadecanedioic acid (OA) and 16-hydroxy hexadecanoic acid (16OHHA) in the serum has been established. The LC-MS/MS method showed high accuracy and rapidly separates all the lipids within 6 min. Subsequently, the serum concentrations of these lipids in the discovery and test set were determined. The clinical value of these serum lipids in early screening and diagnosis of HCC in the T2DM population was evaluated.

## 2. Materials and Methods

### 2.1. Chemicals and Reagents

High-performance liquid chromatography (HPLC)-grade acetonitrile and isopropanol were from Fisher Chemical. Ammonium acetate was purchased from Aladdin. 8,15-DiHETE and DHK-PGA2 were from Cayman Chemical, RCL was from Alta Scientific, OA and 16OHHA were from Tokyo Chemical Industry, and HDA was from Sigma-Aldrich. The isotopically-labeled HDA-d28 was purchased from ZZ Standards.

### 2.2. Study Population

In the present study, we recruited 482 individuals, including patients with HCC, pancreatic cancer (PC), gastric cancer (GC), colorectal cancer (CRC), and T2DM, as well as normal controls (NCs) from Peking University People’s Hospital, Beijing, China. All participants were divided into discovery and test sets. The discovery set contained 32 T2DM, as well as 19 T2DM(+) HCC individuals. It was used for untargeted metabolomic analyses, from which differential lipids were screened. The test set contained 94 NC, 96 T2DM, 64 T2DM(+) HCC, 90 T2DM-negative [T2DM(−)] HCC, 44 T2DM(+) CRC, 21 T2DM(+) GC and 22 T2DM(+) PC individuals. It was used to validate the results of untargeted metabolomic analyses and explore the diagnostic performance of the differential lipids.

The diagnostic criteria for T2DM and HCC have been described before [9,20]. All HCC, PC, GC, and CRC cases were newly diagnosed patients without treatment and pathologically confirmed by experienced physicians. The clinical stage of HCC patients was determined according to the China liver cancer (CNLC) staging system [20]. Patients with HCC after treatment, as well as those with any other malignancy, were excluded. NC subjects were healthy people undergoing routine examinations in our hospital. Collect peripheral blood from each participant after fasting. The clinical features of the included subjects were collected and summarized in Table 1.

### 2.3. Measurement of Clinical Parameters

Peripheral blood was collected and serum was then separated by centrifugation. Fasting blood glucose (FBG) was measured using AU5832 (Beckman Coulter). Serum AFP was detected by Cobas e801 (Roche Diagnostics). Both tests were performed using the original reagents from the instrument manufacturers according to the standard operating instructions.

### 2.4. Sample Preparation

A mixed standard stock solution comprising 8,15-DiHETE, HDA, DHK-PGA2, RCL, OA, and 16OHHA was prepared to generate calibration curves. Select twenty serum samples randomly and mix these samples equally, and calibrators were serially diluted (2-fold steps) with the pooled serum. The concentrations of the calibrators were determined according to our pre-experiments. Additionally, prepare two quality control (QC) samples (low QC and high QC) by spiking suitable amounts of 8,15-DiHETE, HDA, DHK-PGA2, RCL, OA, and 16OHHA into the mixed serum.

Next, the calibrators, QC, and serum samples were subjected to pretreatment. At first, mix each sample with a two-fold volume of precipitant solution (acetonitrile: methanol = 50:50, containing isotopically-labelled HDA-d28), and centrifuge at 12000 rpm for 10 min at 4 ℃ to precipitate serum proteins. Then transfer the supernatant of each sample into a fresh tube and add a two-fold volume of diluent solution (acetonitrile: H_2_O = 50:50) to dilute the supernatant. Five microliters of each sample were loaded onto the LC-MS/MS platform for quantitative analysis.

### 2.5. LC-MS/MS Analysis

The LC-MS/MS platform was a Jasper HPLC connected with a Triple Quadrupole 4500MD (SCIEX). An Xbridge BEH C8 column (2.1 mm × 100 mm, 2.5 μm, Waters) was applied for metabolite separation. The mobile phase A was 5 mmol/L ammonium acetate in H_2_O-acetonitrile 50/50, and the mobile phase B was 5 mmol/L ammonium acetate in isopropanol-acetonitrile 20/80. The temperature of the auto-sampler and column oven were 8 and 45 °C, respectively. The gradient elution program was: 0–1 min, 100% A; 1–2.5 min, 100%−90% A; 2.5–2.6 min, 90%–0% A; 2.6–4.5 min, 0% A; 4.5–5 min, 0%−100% A; 5–6 min, 100% A. The MS detection was performed using negative electron spray ionization mode. The optimization of multiple reaction monitoring (MRM) transitions of the serum lipids was conducted individually (Supplementary Table S1). The ion spray voltage and capillary temperature were −4500 V and 400 ℃, respectively. The curtain gas and collision gas were 25 and 9 arbitrary units, respectively.

### 2.6. Method Validation

Calibration curves were drawn by plotting the metabolite peak area ratios (analyte/internal standard) of the calibrators minus that of the serum matrix, versus the theoretical concentration of each calibrator. Linear regression analysis with 1/x weighing was employed. The deviation of each concentration point from the theoretical value should be within 15%. Low and high QC were used for precision verification. Analyte recovery was evaluated at three different levels by spiking suitable amounts of analytes into the mixed serum. Carryover was evaluated by measuring the blank sample after the highest point of the calibration curve.

### 2.7. Statistical Analysis

GraphPad Prime 5.01 (GraphPad Software), Microsoft Excel 2021 (Microsoft), or SPSS 20.0 software (IBM) was applied for data analysis. The variables were displayed as mean with standard deviation (SD) or median with interquartile range. A Students *t*-test and Mann-Whitney U test were applied to compare the data with normal and skewed distribution, respectively. Spearman’s correlation analysis was performed to evaluate the correlation between indicators. The receiver operating characteristic (ROC) curve was drawn and the area under the ROC curve (AUC) was calculated. The cut-off point was determined by Youden’s index. The efficacy of the combined diagnosis of different indicators was evaluated by binary logistic regression.

## 3. Results

### 3.1. Method Development and Validation

The ionization and fragmentation of the lipids were measured by injecting each standard into the MS/MS platform using a syringe pump. The MS/MS conditions, such as collision energy, declustering potential, source temperature, and ion-spray voltage, were optimized to achieve high detection sensitivity and specificity of each analyte. Finally, the negative electrospray ionization MRM mode with precursor ion [M-H] and their product ions were used as quantitative transitions (Supplementary Table S1).

**Figure 1:**
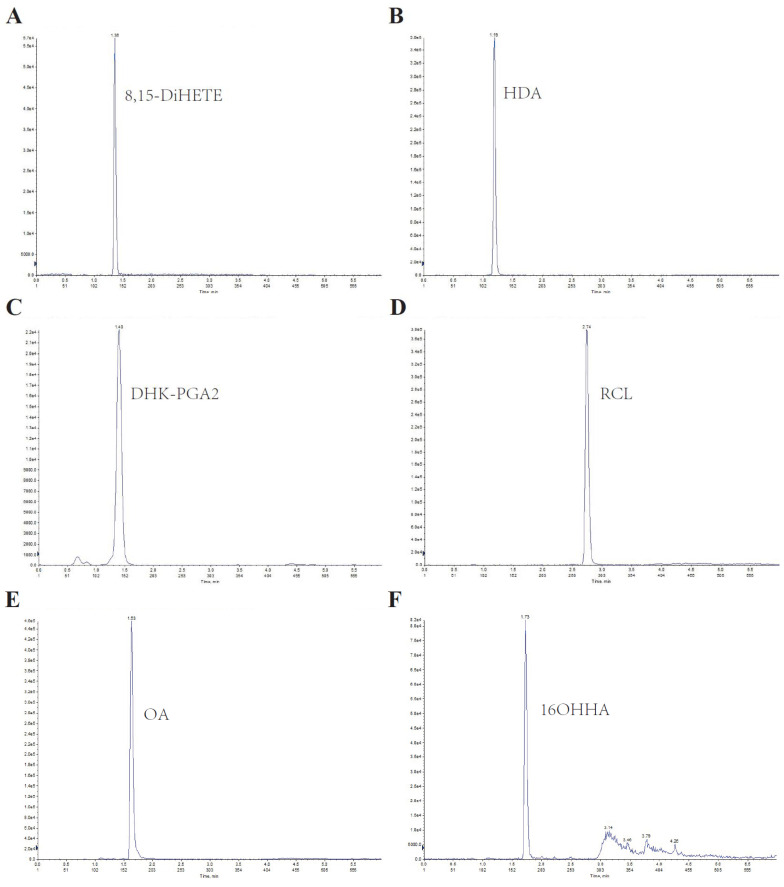
Representative chromatography of the LC–MS/MS method for serum lipids. (**A**–**F**) represent the chromatography of 8,15-DiHETE, HDA, DHK-PGA2, RCL, OA, and 16OHHA, respectively.

Since lipids are hydrophobic, an Xbridge BEH C8 column was used for separation. A gradient program was introduced to elute the lipids separately, and ammonium acetate was added to improve detection sensitivity. The six analytes were effectively separated with good peak shapes, and no significant carryover was observed. The representative chromatographic peak of each serum lipid was displayed in Figure 1.

Then, the method validation was carried out to evaluate the linearity, linear range, precision, and recovery of the above analytical method (Table 2). The linear r^2^ for each calibration curve was >0.99, suggesting a great linearity for all of the serum lipids. The intra- and inter-day imprecisions were evaluated using low and high QC samples, and the coefficient variations (CVs) were <10%. Recovery was assessed at three different concentrations, and the recovery rate ranged from 85% to 105%, indicating the high accuracy of this analytical method. These results suggested that this LC-MS/MS method for 8,15-DiHETE, HDA, DHK-PGA2, RCL, OA, and 16OHHA is reliable.

### 3.2. Validation of Dysregulated Lipids by Targeted LC-MS/MS Analyses

**Figure 2:**
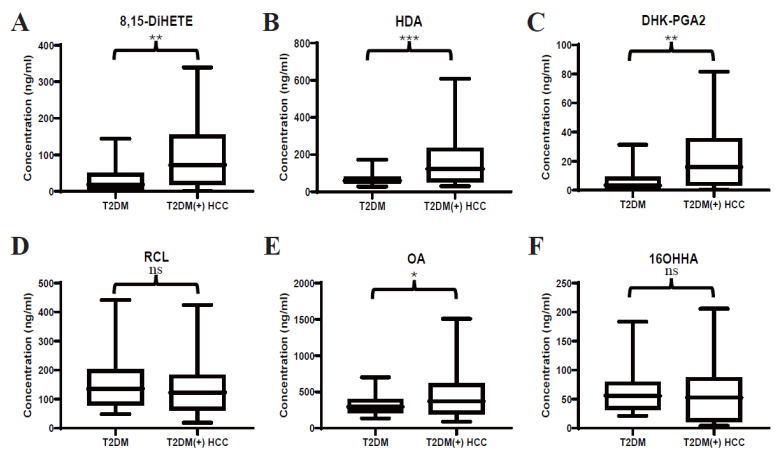
Expression profiles of several dysregulated serum lipids in the discovery set. (**A**–**F**) represent the levels of serum 8,15-DiHETE, HDA, DHK-PGA2, RCL, OA and 16OHHA in the discovery set, respectively. Student’s *t* test or Mann-Whitney U test was applied. T2DM, type 2 diabetes mellitus; HCC, hepatocellular cancer. *** *p* value < 0.001; ** *p* value < 0.01; * *p* value < 0.05; ns, not significant.

In our previous study, several serum lipids were identified to be dysregulated in T2DM(+) HCC patients (Table 3). Here, targeted LC-MS/MS analyses were performed to validate the previous untargeted metabolomic profiling and precisely quantify the levels of 8,15-DiHETE, HDA, DHK-PGA2, RCL, OA, and 16OHHA in serum. Consistently, the levels of 8,15-DiHETE (Figure 2A), HDA (Figure 2B), DHK-PGA2 (Figure 2C), and OA (Figure 2E) were significantly upregulated in T2DM(+) HCC in the discovery set. However, the levels of RCL (Figure 2D) and 16OHHA (Figure 2F) were statistically comparable in the two groups.

**Figure 3:**
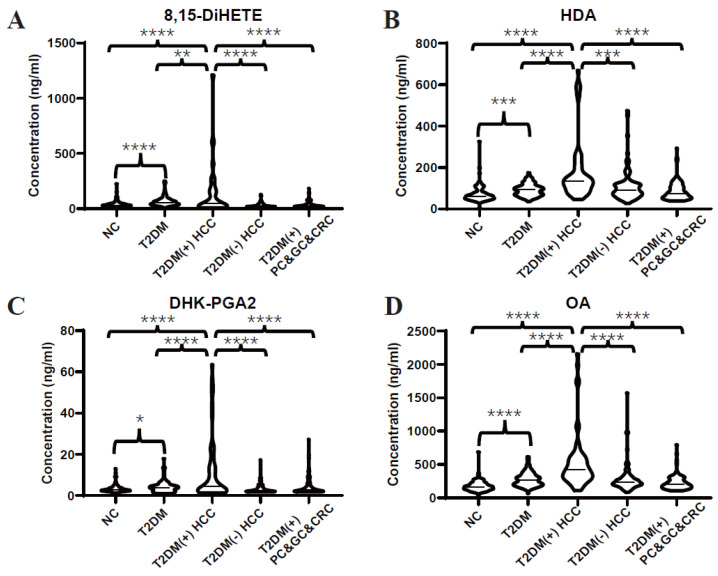
Expression profiles of several dysregulated serum lipids in the test set. (**A**–**D**) represent the levels of serum 8,15-DiHETE, HDA, DHK-PGA2 and OA in the test set, respectively. Student’s *t* test or Mann-Whitney U test was applied. NC, normal control; T2DM, type 2 diabetes mellitus; HCC, hepatocellular cancer; CRC, colorectal cancer; PC, pancreatic cancer; GC, gastric cancer. **** *p* value < 0.0001; *** *p* value < 0.001; ** *p* value < 0.01; * *p* value < 0.05.

Subsequently, an independent test set was included to further validate the dysregulated lipids. Consistently, the differential expressions of 8,15-DiHETE (Figure 3A), HDA (Figure 3B), DHK-PGA2 (Figure 3C), and OA (Figure 3D) between T2DM(+) HCC and T2DM individuals were found in the test set as well. Additionally, T2DM(+) HCC patients showed higher levels of these serum lipids than patients with T2DM(-) HCC and other T2DM(+) cancers, including GC, PC, and CRC, indicating that these lipids were specifically elevated in T2DM(+) HCC.

### 3.3. Evaluation of the Diagnostic Efficacy of the Targeted Lipids

**Figure 4:**
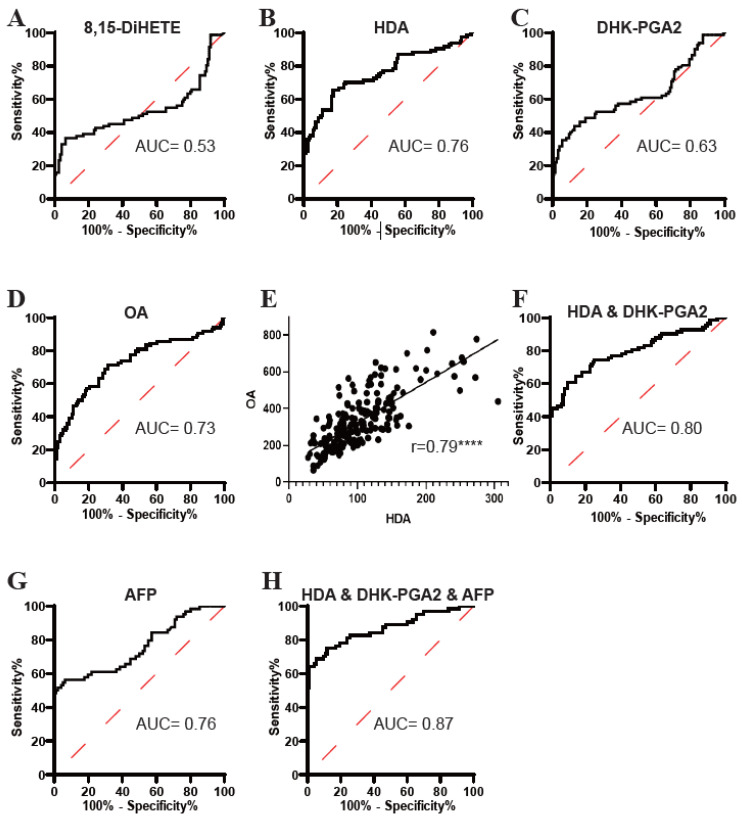
The diagnostic performance of 8,15-DiHETE, HDA, DHK-PGA2 and OA in the test set. (**A**) The ROC curve of 8,15-DiHETE for discriminating T2DM(+) HCC from T2DM patients. (**B**) The ROC curve of HDA for discriminating T2DM(+) HCC from T2DM patients. (**C**) The ROC curve of DHK-PGA2 for discriminating T2DM(+) HCC from T2DM patients. (**D**) The ROC curve of OA for discriminating T2DM(+) HCC from T2DM patients. (**E**) Linear correlation of HDA and OA levels in T2DM and T2DM(+) HCC patients in the test set. Spearman’s correlation analysis was employed. **** *p* value < 0.0001. (**F**) The ROC curve of the combination of HDA and DHK-PGA2 for discriminating T2DM(+) HCC from T2DM patients. (**G**) The ROC curve of AFP for discriminating T2DM(+) HCC from T2DM patients. (**H**) The ROC curve of the biomarker signature based on HDA, DHK-PGA2 and AFP for discriminating T2DM(+) HCC from T2DM patients. AFP, alpha-fetoprotein; T2DM, type 2 diabetes mellitus; HCC, hepatocellular cancer; ROC, receiver operating characteristic; AUC, area under the ROC curve.

Subsequently, the diagnostic potential of 8,15-DiHETE, HDA, DHK-PGA2, and OA was evaluated by ROC analyses. As displayed in Figure 4, the AUC of 8,15-DiHETE in distinguishing T2DM(+) HCC from T2DM patients was low (Figure 4A), while the AUCs of HDA (Figure 4B), DHK-PGA2 (Figure 4C) and OA (Figure 4D) were acceptable. Then, 8,15-DiHETE was excluded in the following analysis because of its poor diagnostic performance. Next, the correlations between HDA, DHK-PGA2, and OA were evaluated, and we found that HDA was closely correlated with OA (r = 0.79) (Figure 4E), while the correlation between OA and DHK-PGA2 (Supplementary Figure S1A), as well as that between HDA and DHK-PGA2 (Supplementary Figure S1B), was not significant. As HDA was closely correlated with OA, their combination did not improve the diagnostic performance (AUC = 0.76) compared to HDA alone (Supplementary Figure S1C). Therefore, OA was excluded and the diagnostic efficacy of the combination of HDA and DHK-PGA2 was evaluated. The AUC of HDA&DHK-PGA2 was 0.80, with an optimal sensitivity of 60.98% and specificity of 90.24% (Figure 4F, Table 4).

Because the diagnostic performance of AFP was unsatisfactory (Figure 4G, Table 4), we then evaluated whether the cooperation of HDA&DHK-PGA2 and AFP could further elevate the diagnostic efficacy for T2DM(+) HCC. A logistic regression model based on HDA, DHK-PGA2 and AFP was constructed: logit [p = HCC] = 0.022 × [HDA] + 0.10 × [DHK-PGA2] + 0.219 × [AFP] − 4.816. As displayed in Figure 4H and Table 4, the biomarker signature displayed better efficacy than HDA&DHK-PGA2 and AFP individually (AUC 0.87, optimal sensitivity 68.75%, and specificity 94.62%). Taken together, these data suggested the great performance of the biomarker signature that incorporated HDA, DHK-PGA2, and AFP for T2DM(+) HCC monitoring in T2DM individuals.

### 3.4. Clinical Value of the Biomarker Signature for Small-Size and Early-Stage T2DM(+) HCC

**Figure 5:**
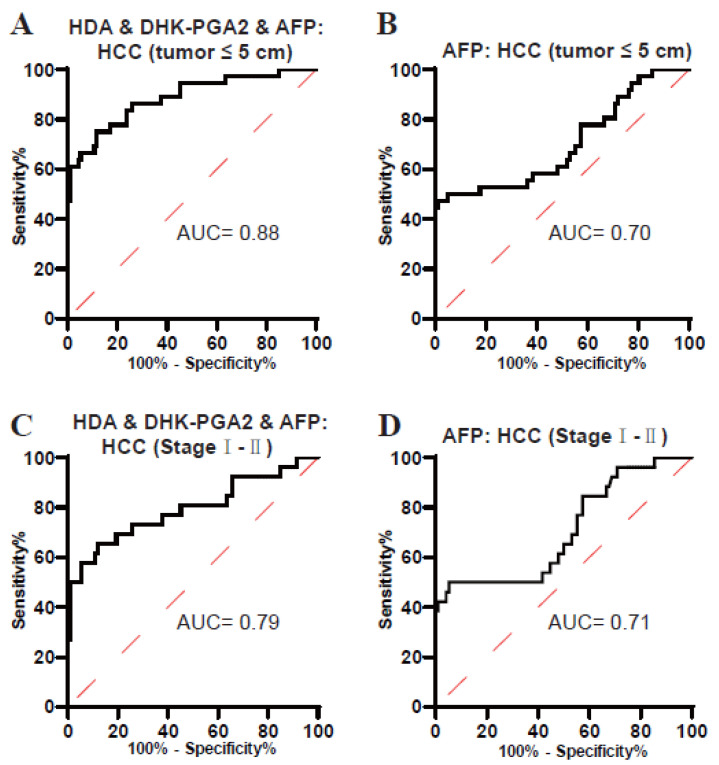
The role of the biomarker signature in the diagnosis of small-size and early-stage T2DM(+) HCC patients. (**A**) The ROC curve of the biomarker signature for discriminating T2DM(+) HCC patients with tumors ≤ 5 cm from T2DM patients. (**B**) The ROC curve of AFP for discriminating T2DM(+) HCC patients with tumors ≤ 5 cm from T2DM patients. (**C**) The ROC curve of the biomarker signature for discriminating T2DM(+) HCC patients with stageI-IItumors from T2DM patients. (**D**) The ROC curve of AFP for discriminating T2DM(+) HCC patients with stageI-II tumors from T2DM patients. AFP, alpha-fetoprotein; T2DM, type 2 diabetes mellitus; HCC, hepatocellular cancer; ROC, receiver operating characteristic; AUC, area under the ROC curve.

It is well known that small-size and early-stage HCCs are usually not easy to diagnose. Next, the performance of the biomarker signature in the diagnosis of these tumors was evaluated. As displayed in Figure 5A and Table 5, the AUC of the biomarker signature was 0.88 in diagnosing small-size T2DM(+) HCC (the optimal sensitivity was 75.00% and a specificity of 88.17%). In addition, the AUC of the biomarker signature was 0.79 in diagnosing early-stage T2DM(+) HCC (the optimal sensitivity was 65.38% and a specificity of 88.17%) (Figure 5C, Table 5). However, the diagnostic efficacy of AFP was much poorer than the biomarker signature in differentiating small-size and early-stage T2DM(+) HCC from T2DM (Figure 5B,D, Table 5). Collectively, these data suggested the clinical value of the biomarker signature for small-size and early-stage T2DM(+) HCC.

### 3.5. The Diagnostic Specificity of the Biomarker Signature

**Figure 6:**
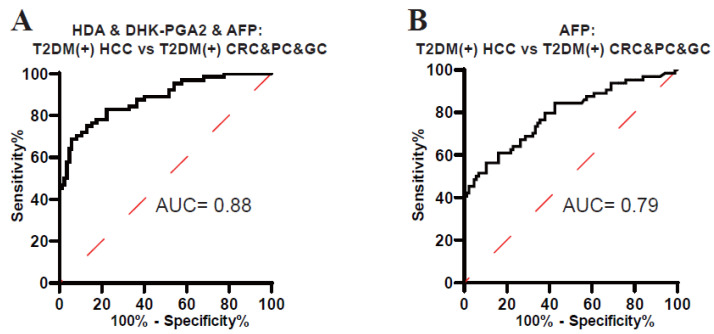
The specificity of the biomarker signature in the diagnosis of T2DM(+) HCC patients. (**A**) The ROC curve of the biomarker signature for discriminating T2DM(+) HCC from T2DM(+) CRC&PC&GC patients. (**B**) The ROC curve of AFP for discriminating T2DM(+) HCC from T2DM(+) CRC&PC&GC patients. T2DM, type 2 diabetes mellitus; HCC, hepatocellular cancer; CRC, colorectal cancer; PC, pancreatic cancer; GC, gastric cancer; ROC, receiver operating characteristic; AUC, area under the ROC curve.

As displayed in Figure 3, HDA and DHK-PGA2 were specifically elevated in T2DM(+) HCC, but not in T2DM(+) CRC, T2DM(+) GC, or T2DM(+) PC, indicating the high specificity of our biomarker signature for T2DM(+) HCC. To validate its high specificity, the ROC analysis was conducted, and the AUC of the biomarker signature was 0.88 to diagnose T2DM(+) HCC from T2DM(+) CRC&PC&GC (the optimal sensitivity 68.75% and specificity 94.12%), which was much higher than AFP (Figure 6, Table 6). Altogether, these results confirmed the high specificity of the signature for T2DM(+) HCC.

## 4. Discussion

In this study, a robust LC-MS/MS method was developed for the simultaneous measurement of several serum lipids, including 8,15-DiHETE, HDA, DHK-PGA2, RCL, OA, and 16OHHA. Using this LC-MS/MS method, we found that serum 8,15-DiHETE, HDA, DHK-PGA2, and OA were significantly upregulated in T2DM(+) HCC patients. A biomarker signature based on HDA, DHK-PGA2, and AFP showed great diagnostic efficacy in diagnosing T2DM(+) HCC from T2DM and other T2DM(+) cancers, including GC, PC, and CRC, suggesting its potential for clinical application. With the increasing popularity of the LC-MS/MS platform in Chinese hospitals, as well as the increasing number of LC-MS/MS-related training courses and technicians skilled in LC-MS/MS, this simple method could be easily and widely used in other laboratories. As the number of T2DM patients is growing globally and the association between T2DM and HCC is tight, it is increasingly essential to discover potential tumor markers for HCC monitoring in T2DM individuals [21,22]. There is still a lack of good biomarkers for T2DM(+) HCC monitoring at present, and how to screen HCC tumors, especially those with small size and early stage, from T2DM individuals is still a challenge. In this study, the diagnostic performance of the classical biomarker AFP was unsatisfactory, especially for small-size and early-stage HCC, which was consistent with the previous studies [4,12]. Here, we constructed a biomarker signature including HDA, DHK-PGA2, and AFP. The biomarker signature was shown to efficiently differentiate T2DM(+) HCC from T2DM patients, and it detected small-size and early-stage HCC effectively. This study highlights the clinical value of the biomarker signature in the detection of T2DM(+) HCC, which has critical implications for improving outcomes for patients with T2DM(+) HCC.

Although numerous studies have suggested a close relationship between HCC and T2DM, how T2DM-induced HCC tumorigenesis is still largely unknown. It was reported that insulin resistance contributes significantly to HCC carcinogenesis by regulating oxidative stress, gut microbiota, and the production of proinflammatory mediators [23]. In addition, several signaling pathways, such as vascular endothelial growth factor (VEGF) and insulin-like growth factor (IGF) pathway, play roles in the development of diabetes-related HCC [22]. Moreover, it has been suggested that several metabolites were important for the development of T2DM(+) HCC as well. For example, 2-hydroxystearate was upregulated in T2DM(+) HCC and might have a critical function in HCC development in T2DM individuals [24]. In our previous study, several amino acids and their metabolic products were dysregulated in T2DM(+) HCC significantly, confirming the important role of small molecular metabolites in hepatocarcinogenesis in T2DM patients [19].

The abnormal lipid metabolism is one of the most significant characteristics of cancer and has important roles in a lot of biological processes, including cancer cell proliferation, metastasis, invasion, and survival [25,26]. The elevation of lipogenic enzymes, including ATP-citrate lyase (ACLY) and acetyl-CoA synthetase 2 (ACSS2), resulted in increased fatty acid synthesis and is often associated with poorer prognosis in cancer patients [27,28]. In addition, it is emerging that lipid metabolism regulates the oncogenic EGFR signaling pathway [29,30]. Previous studies have suggested the potential of dysregulated lipids in the diagnosis of cancer as well. For example, it has been reported that many lipids, such as phosphatidylcholine, triglyceride, diacylglycerol, phosphatidylinositol, and eicosanoids were altered in HCC, and showed great potential for HCC diagnosis [31,32,33,34,35]. In this study, several serum lipids, such as HDA and DHK-PGA2, were also found to be altered in T2DM(+) HCC. HDA is a kind of alkanedioic acid enriched in the human liver and peripheral blood. It might be used to evaluate drug-drug interactions mediated by hepatic drug transporters [36]. DHK-PGA2 is produced via dehydration of 13,14-dihydro-15-ketoprostaglandin E2, which is generally considered to have the potential to promote tumor progression [37]. Consistently, this study described the enrichment of HDA and DHK-PGA2 in human serum and elucidated the considerable clinical values of HDA and DHK-PGA2 for T2DM(+) HCC.

There are still some limitations in this study. At first, this was a single-center study with a small sample size. In addition, the participants we included were mainly from northern China, and our results still need to be verified in other populations due to the regional differences. Moreover, we could not exclude the influence of other risk factors for HCC, such as chronic hepatitis B virus (HBV) and hepatitis C virus (HCV) infection, alcoholic hepatitis, and non-alcoholic fatty liver disease.

In conclusion, the present study established a robust LC-MS/MS method for simultaneously detecting 8,15-DiHETE, HDA, DHK-PGA2, RCL, OA, and 16OHHA, and it is the first to report that serum 8,15-DiHETE, HDA, DHK-PGA2 and OA were elevated in T2DM(+) HCC, and the biomarker signature based on HDA, DHK-PGA2 and AFP shows great diagnostic efficacy for T2DM(+) HCC. Due to the increasing number of T2DM patients and the close relationship between T2DM and HCC, the biomarker signature could be used in the monitoring and early diagnosis of HCC in high-risk T2DM individuals. In the future, it will be still essential to perform multicenter prospective studies to confirm the diagnostic potential of this biomarker signature.

## Figures and Tables

**Table 1:** The clinical features of individuals included in this study.

	Discovery Set		Test Set						
**Variables**	T2DM	T2DM(+) HCC	NC	T2DM	T2DM(+) HCC	T2DM(−) HCC	T2DM(+) CRC	T2DM(+) PC	T2DM(+) GC
	*N* = 32	*N* = 19	*N* = 94	*N* = 96	*N* = 64	*N* = 90	*N* = 44	*N* = 22	*N* = 21
**Age**	56.47 ± 11.37	64.32 ± 9.21	43.56 ± 15.02	55.15 ± 12.92	61.67 ± 9.15	56.88 ± 12.68	68.55 ± 9.99	68.86 ± 8.13	67.90 ± 11.61
**Gender Male/Female**	20/12	17/2	32/62	65/31	54/10	70/20	27/17	15/7	14/7
**FBG (mmol/L)**	7.43 ± 1.43	9.75 ± 4.94	4.99 ± 0.42	8.17 ± 2.37	8.30 ± 3.45	5.07 ± 0.56	7.65 ± 2.01	7.93 ± 2.45	8.85 ± 4.34
**AFP >7/≤7 ng/mL**	3/29	11/8	2/92	6/90	37/27	55/35	2/42	3/19	4/17

**Abbreviations:** T2DM, type 2 diabetes mellitus; HCC, hepatocellular cancer; NC, normal control; CRC, colorectal cancer; PC, pancreatic cancer; GC, gastric cancer; FBG, fasting blood glucose; AFP, alpha-fetoprotein.

**Table 2:** The results of methodology validation for the quantification of serum.8,15-DiHETE, HDA, DHK-PGA2, RCL, OA, and 16OHHA.

	Precisions				Linear Range (ng/mL)	Regression Coefficient (R^2^)	Recoveries		
	Intra-Day		Inter-Day						
	Low	High	Low	High			Low	Median	High
**8,15-DiHETE**	2.05%	1.91%	1.72%	7.05%	3.125–1600	≥0.9989	96.83%	96.45%	92.08%
**HDA**	2.45%	3.05%	1.84%	2.52%	6.25–800	≥0.9990	103.71%	102.70%	104.40%
**DHK-PGA2**	1.31%	2.87%	3.86%	9.49%	0.78125–200	≥0.9982	98.93%	97.40%	93.00%
**RCL**	1.27%	0.30%	1.45%	7.42%	6.25–800	≥0.9992	97.04%	95.42%	91.87%
**OA**	2.25%	1.34%	2.37%	5.21%	25–3200	≥0.9981	97.62%	94.36%	86.45%
**16OHHA**	1.87%	2.25%	2.02%	5.31%	3.125–400	≥0.9989	95.98%	94.95%	93.88%

**Table 3:** List of differential lipids identified in the untargeted metabolomic analyses.

Metabolite	MS2 Score	VIP	*p* Value	FC	Log_FC
**Pregnanetriol**	0.7622	2.0363	0.0000	0.4133	−1.2746
**PC(18:4(6Z,9Z,12Z,15Z)/18:1(11Z))**	0.7082	1.4334	0.0136	0.6198	−0.6901
**N-Cyclopropyl-trans-2-cis-6-nonadienamide**	0.9400	2.7859	0.0000	0.6872	−0.5411
**6beta-Hydroxytestosterone**	0.8066	1.2485	0.0389	0.7675	−0.3817
**PC(22:5(7Z,10Z,13Z,16Z,19Z)/18:2(9Z,12Z))**	0.7928	1.2568	0.0256	0.7891	−0.3417
**PC(22:5(7Z,10Z,13Z,16Z,19Z)/16:0)**	0.6836	1.5313	0.0114	0.8529	−0.2296
**Pelargonic acid**	0.9999	1.2747	0.0178	1.1232	0.1676
**PC(22:5(7Z,10Z,13Z,16Z,19Z)/P-16:0)**	0.8313	1.0545	0.0290	1.3449	0.4275
**(E)-2,6-Dimethyl-2,5-heptadienoic acid**	0.6134	1.5895	0.0005	1.3755	0.4600
**PC(20:3(8Z,11Z,14Z)/20:1(11Z))**	0.8495	1.0410	0.0146	1.3959	0.4812
**PC(P-18:1(11Z)/22:5(4Z,7Z,10Z,13Z,16Z))**	0.8300	1.2593	0.0026	1.3976	0.4829
**PC(P-16:0/16:0)**	0.6759	1.6758	0.0073	1.4797	0.5653
**Azelaic acid**	0.6424	1.3805	0.0056	1.4960	0.5812
**PC(P-18:0/22:4(7Z,10Z,13Z,16Z))**	0.8381	1.7603	0.0004	1.5029	0.5878
**1-O-Hexadecyl-2-O-dihomogammalinolenoylglycero-3-phosphocholine**	0.8612	1.9964	0.0010	1.6454	0.7184
**PC(18:2(9Z,12Z)/P-18:1(11Z))**	0.7329	1.7922	0.0003	1.6477	0.7204
**3-Hydroxyisovaleric acid**	0.9370	1.7349	0.0001	1.6689	0.7389
**Ethyl oleate**	0.7968	1.1517	0.0072	1.6872	0.7546
**PC(18:0/P-16:0)**	0.6660	2.3506	0.0003	1.7399	0.7990
**16-Hydroxy hexadecanoic acid**	0.9732	1.8678	0.0020	2.0424	1.0302
**Octadecanedioic acid**	0.6481	1.0454	0.0340	2.1652	1.1145
**Ricinoleic acid**	0.9862	1.4726	0.0454	2.6921	1.4288
**15-Keto-13,14-dihydroprostaglandin A2**	0.8173	1.6023	0.0457	2.7710	1.4704
**Hexadecanedioic acid**	0.9007	2.0187	0.0158	5.2489	2.3920
**8,15-DiHETE**	0.9765	1.5252	0.0114	7.9630	2.9933

**Abbreviations:** VIP, variable importance in the projection; FC, fold change.

**Table 4:** The diagnostic performance of the following indicator and their combination for the detection of T2DM(+) HCC from the T2DM population.

	AUC (95%CI)	Sensitivity (%)	Specificity (%)	*p*-Value
8,15-DiHETE	0.53 (0.44–0.62)	39.56	93.60	0.4534
HDA	0.76 (0.67–0.83)	65.48	82.40	<0.0001
DHK-PGA2	0.63 (0.55–0.71)	43.90	87.30	0.0014
OA	0.73 (0.65–0.80)	71.43	68.75	<0.0001
HDA & DHK-PGA2	0.80 (0.73–0.86)	60.98	90.24	<0.0001
AFP	0.76 (0.68–0.84)	56.25	93.75	<0.0001
HDA & DHK-PGA2 & AFP	0.87 (0.80–0.93)	68.75	94.62	<0.0001

**Table 5:** The diagnostic performance of the biomarker signature for the detection of small-size and early-stage T2DM(+) HCC.

Groups		AUC (95%CI)	Sensitivity (%)	Specificity (%)	*p*-Value
HCC (≤5 cm) vs. T2DM					
	**AFP**	0.70 (0.59–0.81)	47.22	98.96	0.0004
	**The Biomarker Signature**	0.88 (0.81–0.95)	75.00	88.17	<0.0001
HCC (I-II) vs. T2DM					
	**AFP**	0.71 (0.58–0.83)	50.00	94.79	0.0013
	**The Biomarker Signature**	0.79 (0.68–0.91)	65.38	88.17	<0.0001

**Table 6:** The diagnostic specificity of the biomarker signature for the detection of T2DM(+) HCC from T2DM(+) CRC&PC&GC.

	AUC (95%CI)	Sensitivity (%)	Specificity (%)	*p*-Value
**AFP**	0.79(0.72–0.87)	56.25	89.66	<0.0001
**The Biomarker Signature**	0.88(0.83–0.94)	68.75	94.12	<0.0001

## Data Availability

The original contributions presented in the study are all included in the article/Supplementary Material.

## Supplementary Materials

The following supporting information can be downloaded at: https://www.mdpi.com/article/10.3390/metabo13010090/s1, Figure S1: (A) Spearman’s correlation analysis of DHK-PGA2 and OA levels in T2DM and T2DM(+) HCC patients in the test set. (B) Spearman’s correlation analysis of DHK-PGA2 and HDA levels in T2DM and T2DM(+) HCC patients in the test set. (C) The ROC curve of the combination of HDA and OA for discriminating T2DM(+) HCC from T2DM patients; Table S1: MRM settings for 8,15-DiHETE, HDA, DHK-PGA2, RCL, OA and 16OHHA.
