# Supplementary material for: Simultaneous Quantification of Serum Lipids and Their Association with Type 2 Diabetes Mellitus-Positive Hepatocellular Cancer
Source: Metabolites. 2023 Jan 6;13(1):90. doi: 10.3390/metabo13010090 (PMC9865394; doi:10.3390/metabo13010090)
Supplement: Supplementary file 1 [file metabolites-13-00090-s001.zip › metabolites-2077108-Supplementary Table S1.pdf]

Supplementary Table S1. MRM settings for 8,15-DiHETE, HDA, DHK-PGA2, RCL, OA and 16OHHA

Q1 Mass (Da)	Q3 Mass (Da)	Dwell Time (msec)	ID	DP (volts)	EP (volts)	CE (volts)	CXP (volts)
285.1	223.1	30	HDA-1	-90	-10	-34	-15
285.1	221.2	30	HDA-2	-90	-10	-36	-15
285.1	267.2	30	HDA-3	-90	-10	-27	-15
313.2	251.1	30	OA-1	-80	-10	-33	-15
313.2	295.2	30	OA-2	-140	-10	-38	-15
297.2	183	30	RCL-1	-90	-10	-28	-15
297.2	112.7	30	RCL-2	-90	-10	-30	-15
271.2	224.9	30	16OHHA-2	-90	-10	-31	-15
271.2	223.2	30	16OHHA-1	-140	-7	-36	-10
271.2	197.1	30	16OHHA-3	-140	-7	-38	-10
313.4	250.3	30	HDA-d28-1	-70	-10	-35	-8
313.4	269.3	30	HDA-d28-2	-70	-10	-35	-8
333.2	235.1	30	DHK-PGA2-1	-90	-10	-28	-8
333.2	113.1	30	DHK-PGA2-2	-90	-10	-31	-8
335.2	208.1	30	8,15-DiHETE-1	-70	-10	-24	-7
335.2	235.1	30	8,15-DiHETE-2	-70	-10	-22	-7
335.2	190.1	30	8,15-DiHETE-3	-70	-10	-29	-5

Abbreviations: DP, declustering potential; EP, entrance potential; CE, collision energy; CXP, cell exit potential.
