# Supplementary figures and images for: Simultaneous Quantification of Serum Lipids and Their Association with Type 2 Diabetes Mellitus-Positive Hepatocellular Cancer
Source: Metabolites. 2023 Jan 6;13(1):90. doi: 10.3390/metabo13010090 (PMC9865394; doi:10.3390/metabo13010090)

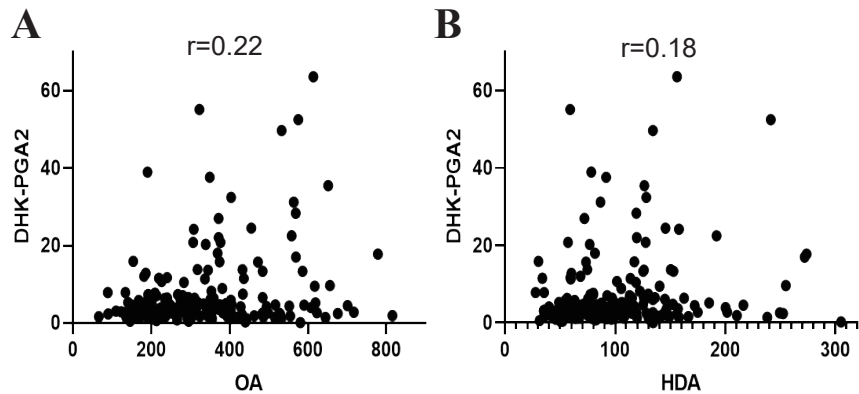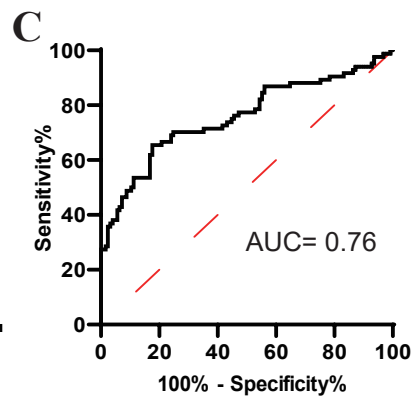

Supplement: Supplementary file 1 [file metabolites-13-00090-s001.zip › metabolites-2077108-Supplementary Figure S1.pdf]
